# Catalytic Asymmetry in Homodimeric H^+^-Pumping Membrane Pyrophosphatase Demonstrated by Non-Hydrolyzable Pyrophosphate Analogs

**DOI:** 10.3390/ijms22189820

**Published:** 2021-09-10

**Authors:** Viktor A. Anashkin, Anssi M. Malinen, Alexander V. Bogachev, Alexander A. Baykov

**Affiliations:** 1 Belozersky Institute of Physico-Chemical Biology, Lomonosov Moscow State University, 119899 Moscow, Russia; victor_anashkin@belozersky.msu.ru (V.A.A.); bogachev@belozersky.msu.ru (A.V.B.); 2 Department of Life Technologies, University of Turku, FIN-20014 Turku, Finland; ansmal@utu.fi

**Keywords:** proton pump, bisphosphonate, etidronate, cooperativity, energy coupling, enzyme kinetics

## Abstract

Membrane-bound inorganic pyrophosphatase (mPPase) resembles the F-ATPase in catalyzing polyphosphate-energized H^+^ and Na^+^ transport across lipid membranes, but differs structurally and mechanistically. Homodimeric mPPase likely uses a “direct coupling” mechanism, in which the proton generated from the water nucleophile at the entrance to the ion conductance channel is transported across the membrane or triggers Na^+^ transport. The structural aspects of this mechanism, including subunit cooperation, are still poorly understood. Using a refined enzyme assay, we examined the inhibition of K^+^-dependent H^+^-transporting mPPase from *Desulfitobacterium hafniensee* by three non-hydrolyzable PP_i_ analogs (imidodiphosphate and C-substituted bisphosphonates). The kinetic data demonstrated negative cooperativity in inhibitor binding to two active sites, and reduced active site performance when the inhibitor or substrate occupied the other active site. The nonequivalence of active sites in PP_i_ hydrolysis in terms of the Michaelis constant vanished at a low (0.1 mM) concentration of Mg^2+^ (essential cofactor). The replacement of K^+^, the second metal cofactor, by Na^+^ increased the substrate and inhibitor binding cooperativity. The detergent-solubilized form of mPPase exhibited similar active site nonequivalence in PP_i_ hydrolysis. Our findings support the notion that the mPPase mechanism combines Mitchell’s direct coupling with conformational coupling to catalyze cation transport across the membrane.

## 1. Introduction

Early forms of life appear to have depended on pyrophosphate (PP_i_) as the primary energy currency instead of ATP—the so-called “PP_i_ world” [1]. Contemporary organisms produce PP_i_ from ATP and other nucleoside triphosphates as a byproduct in numerous biosynthetic reactions [2]. This PP_i_ reenters the metabolism primarily as P_i_ via the action of inorganic pyrophosphatases (PPases), most of which are soluble enzymes that dissipate PP_i_ energy as heat. However, all plants and many prokaryotic organisms have retained a relict membrane PPase (mPPase; EC 7.1.3.1, formerly 3.6.1.1), which uses PP_i_ energy to transport H^+^ and/or Na^+^ ions across membranes [3,4,5,6,7,8]. This reaction is thermodynamically reversible, and there is controversy about the direction in which mPPase works in cells. mPPases are divided into two homologous families based on their K^+^ requirement for activity. All K^+^-independent mPPases are H^+^ transporters, whereas the K^+^-dependent family is more diverse, and includes mPPases that, depending on Na^+^ concentration, can transport either Na^+^ or both Na^+^ and H^+^ [4]. An increasing body of evidence indicates that mPPases contribute to the tolerance of plants and the bacteria that live in harsh conditions to abiotic stress, such as salination, drought, cold, anoxia, and nutrient limitation [9,10,11,12]. 

**Figure 1:**
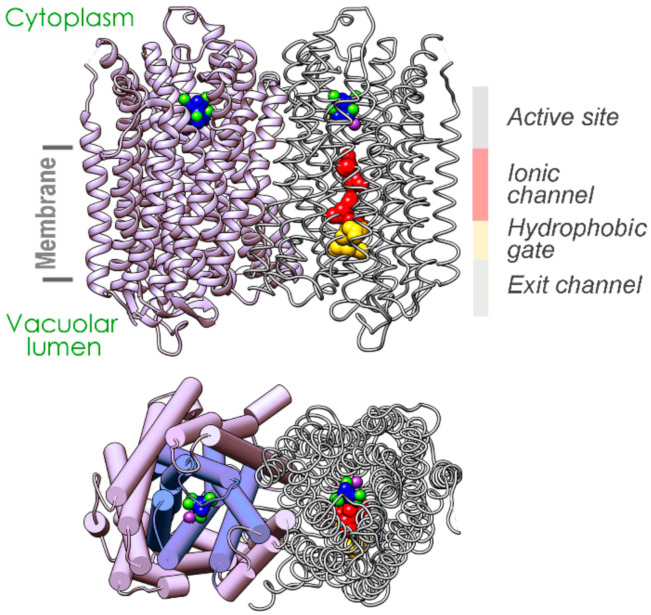
The crystal structure of the *Vigna radiata* membrane pyrophosphatase (mPPase) dimer [17]. The upper panel shows a view along the membrane, whose boundaries are indicated by bars. The substrate analog (imidodiphosphate, IDP), five Mg^2+^ ions, and K^+^ ion bound in the active site are depicted in a space-filling mode (blue, green, and violet, respectively). The residues forming the ionic channel and hydrophobic gate are shown in one subunit in a space-filling node and colored red and yellow. The lower panel shows a view perpendicular to the membrane from the cytoplasmic side. The two concentric rings of the helices are shown as cylinders in one subunit.

mPPase functions in parallel with F-type ATPase, its highly evolved analog, which couples the same transport reactions with ATP synthesis/hydrolysis. Despite this functional similarity, the two transporters have entirely different structures. mPPase is a single 66–89 kDa polypeptide that folds into 15–17 α-helices and forms dimers in the membrane (Figure 1), whereas mitochondrial F-ATPase is formed by 28 polypeptides of 17 different kinds. The transport mechanisms also differ principally. In the F-ATPase, the hydrolysis site and the ion-conducting channel are separated in space. Accordingly, F-ATPase operation in both directions depends on the conformational energy allosterically generated in enzyme molecules by ATP hydrolysis or cation transport in ATP synthesis (“indirect coupling”). The so-called “binding-change” or “rotational mechanism” accepted for this enzyme posits that its three active sites cyclically adopt three conformations with different affinities for the substrate via the rotation of the γ-subunit inside the ring made of the catalytic α- and β-subunits [13,14,15,16]. In mPPase, the water molecule that attacks PP_i_ and releases H^+^ as a byproduct is located just at the entrance to the ion-conducting channel, consisting of the ionic channel, the hydrophobic gate, and the exit channel [17] (Figure 1). Other mPPases are predicted to have very similar structures. That the transported H^+^ ion is the one generated from the nucleophilic water (“direct coupling”) is supported by the finding that H^+^ transport occurs synchronously with PP_i_ hydrolysis [18]. Hypothetically, the same water-generated proton can trigger Na^+^ transport via a “billiards-type” mechanism in mPPase [18]. 

While being principally different, the mPPase mechanism may nevertheless involve elements of F-ATPase conformational coupling, as suggested by the functional nonequivalence of two active sites in the mPPase dimer. The enzyme dimer with PP_i_ bound in both active sites is a less efficient catalyst in terms of both the Michaelis constant and turnover number than the enzyme having PP_i_ in only one active site [19,20]. The H^+^ transport reaction demonstrates a similar dependence on the substrate concentration [20,21]. Additional support for dimer asymmetry comes from earlier radiation inactivation experiments [22,23,24] and the recent observation that *N*-[(2-amino-6-benzothiazolyl)methyl]-1H-indole-2-carboxamide (ATC) abolishes mPPase activity by binding to only one subunit in the dimeric enzyme on the side of the membrane opposite to the side where PP_i_ hydrolysis occurs [25]. It was speculated that mPPase subunits undergo turnover-linked oscillations between two conformations to store conformational energy [19]. The oscillatory mechanism is expected to be less efficient in energy transduction than the rotational mechanism of the F-ATPases, but still might suffice, because PP_i_ hydrolysis releases two times less energy than ATP hydrolysis [2]. 

**Figure 2:**
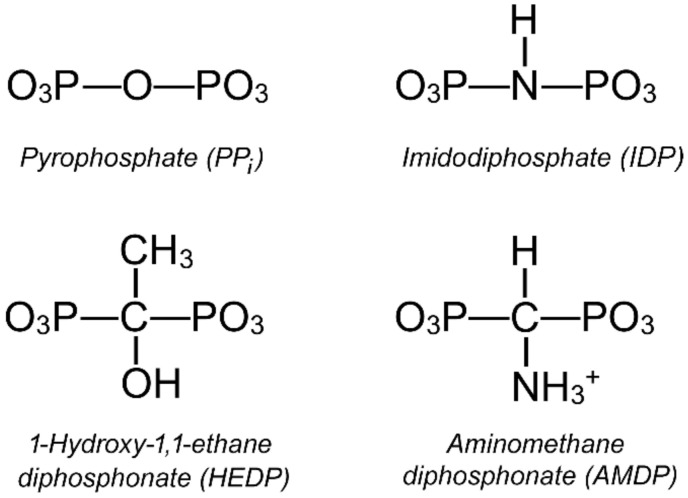
Structures of the PP_i_ analogs. The bridging groups of IDP and AMDP are shown in the ionic forms that prevail at pH 7.2 [26,27]. The phosphate residues are predominantly deprotonated in the magnesium complexes of all analogs.

In this work, we explored the functional asymmetry in the K^+^-dependent H^+^-transporting mPPase of *Desulfitobacterium hafniensee* (Dh-mPPase) by analyzing its inhibition by three non-hydrolyzable PP_i_ analogs (Figure 2). This mPPase is a close homolog of the *V. radiata* mPPase (41% sequence identity), whose structure is shown in Figure 1. Unlike the inhibitor used by Vidilaseris et al. [25], PP_i_ analogs bind to active sites, allowing the measurement of the effects of inhibitor or substrate binding in one subunit on their binding and catalysis in the other subunit. The results support and further extend the concept of active site cooperation in mPPase. 

## 2. Results

### 2.1. Kinetics of Dh-mPPase Inhibition by Substrate Analogs Demonstrates Nonequivalent Active Sites

**Scheme 1:**
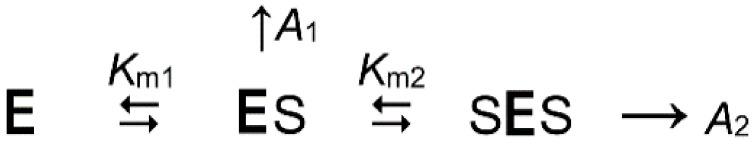
The kinetic model for the reaction catalyzed by the homodimeric mPPase with two interacting active sites at fixed concentrations of K^+^, free Mg^2+^ ions, and substrate analogs. The designations are as follows: E, enzyme dimer; S, the substrate (Mg_2_PP_i_). *K*_m1_ and *K*_m2_ are macroscopic Michaelis constants, *A*_1_ and *A*_2_ are the specific activities of the ES and SES species.

We have earlier demonstrated that the dependence of mPPase activity on Mg_2_PP_i_ concentration is described by a bell-shaped curve with the maximum at approximately 100 µM substrate [19]. This observation was explained in terms of the model shown in Scheme 1, assuming the non-identical behavior of two active sites in the homodimeric mPPase. Specifically, the substrate’s interaction with the second active site is characterized by a much larger (by two orders of magnitude) Michaelis constant and decreased (severalfold) catalytic constant compared to the enzyme having a substrate in only one active site per dimer. 

**Figure 3:**
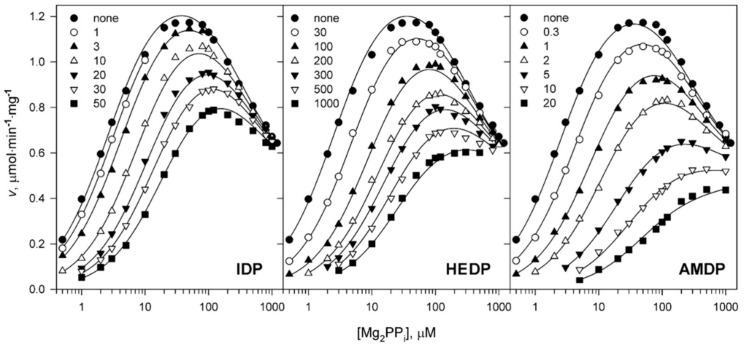
Steady-state kinetics of Dh-mPPase inhibition by substrate analogs. All data were collected with 5 mM Mg^2+^ and 50 mM K^+^ as the alkali metal cofactor. The abscissa shows substrate (Mg_2_PP_i_) concentration and is scaled logarithmically. The total concentrations of substrate analog (in µM) corresponding to different symbols are defined on each panel. Its solubility limited the range of its concentrations used. The lines were calculated using Equation (2) with the parameter values found in Table 1.

Here we confirmed the substrate inhibition of Dh-mPPase (Figure 3) by using a refined assay protocol. Specifically, we considered the PP_i_ interference with P_i_ assay (Figure S1) and avoided the use of tetramethylammonium hydroxide in the buffer preparation after noting that some batches of this alkali supplied in glass containers have an adverse effect on PPase activity [28]. It was also confirmed that the substrate, Mg_2_PP_i_, retains solubility up to 1.2 mM during the activity assay at 25 °C [28]. 

To go further, we explored the binding mechanism for three non-hydrolyzable pyrophosphate analogs containing N or C instead of O in the bridge position (Figure 2). We sought to determine whether these inhibitors also cooperatively bind and affect catalysis in the neighboring subunit. All PP_i_ analogs decreased the maximum activity and shifted the substrate dependence to higher concentrations (Figure 3). The most significant effect was observed with AMPD, the most specific inhibitor of mPPase [29,30]. Analog effects on activity were greatly reduced at the highest substrate concentrations, at which the profiles tended to overlap (Figure 3). This behavior is consistent with a competitive inhibition mechanism. Notably, IDP and AMDP did not affect P_i_ determination at the low concentrations used, whereas HEDP decreased P_i_ analyzer sensitivity by 10% at 1 mM. This effect was proportional to HEDP concentration and is considered in Figure 3. The effects of PP_i_ and HEDP were cumulative.

**Figure 4:**
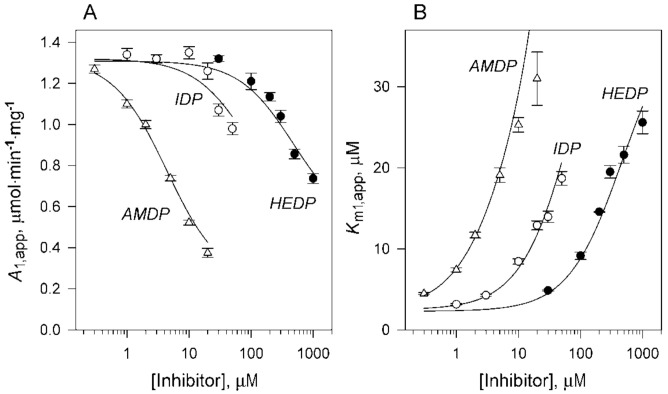
Secondary dependencies of the parameters in Scheme 1 (**A**), maximal activities; (**B**), *K*_m_ values) on the inhibitor concentration, as calculated from the data in Figure 3. The lines were calculated using Equation (2a,c) with the parameter values found in Table 1.

**Scheme 2:**
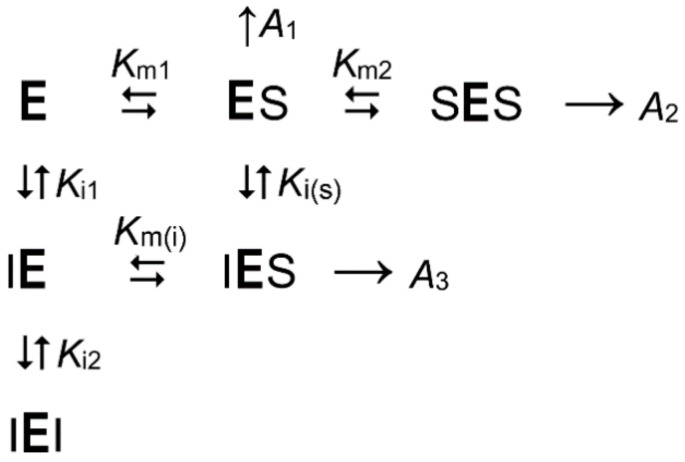
The kinetic model describing the substrate and substrate analog competition for two interacting active sites in mPPase at fixed concentrations of free Mg^2+^ and K^+^ ions. I is an inhibitor (substrate analog); *K*_m1_, *K*_m2_, and *K*_m(i)_ are macroscopic Michaelis constants; *K*_i1_, *K*_i2_, and *K*_i(s)_ are macroscopic inhibitor-binding constants; *A*_1_–*A*_3_ are the specific activities of the corresponding substrate-containing enzyme species.

To identify the enzyme species formed in the presence of the substrate and inhibitor, we analyzed the rate data using Schemes 1 and 2. First, Equation (1) was fitted to each profile to estimate the apparent values of *A*_1_ and *K*_m1_ (Figure 4) that characterize the interaction of the enzyme dimer with the first substrate molecule. The parameters *A*_2_ and *K*_m2_ that refer to the second substrate molecule bound could be estimated with reasonable precision for only low inhibitor concentrations because of difficulties in enzyme saturation with the substrate at high inhibitor concentrations. However, it was clear from this analysis that *A*_2_ is smaller than *A*_1_, and is not zero. Furthermore, the increase in *K*_m1_ in the presence of the inhibitors is consistent with their competition with the substrate. Still, the concomitant decrease in *A*_1_ (Figure 4A) rules out a simple competition mechanism, for which no change in the maximal activity is expected. This effect points to the formation of a mixed complex. Furthermore, it shows that its associated activity (*A*_3_) is less than *A*_1_, because otherwise, *A*_1,app_, according to Equation (2a), would not decrease with an increase in [I].

Therefore, the data were further analyzed using Scheme 2, in which the simple competition of substrate and inhibitor is supplemented with the formation of a catalytically active mixed enzyme–substrate–inhibitor complex (IES). The global fitting of Equation (2) to the rate data indicated that, despite many parameters, there is only one stable set of parameters for each inhibitor (Table 1). *K*_i2_ for IDP and HEDP was the only exception, because it could be set to any value above the indicated limits without an effect on fit quality, as characterized by the root-mean-square deviation (RMSD) of the rate data. This finding indicates that the second molecule of these inhibitors binds very weakly. In contrast, setting *K*_i2_ for AMDP to infinity made the fit significantly worse (Table S1), indicating the formation of the IEI complex. This deduction explains the marked effect of AMDP, but not of the other inhibitors, at the highest substrate concentrations in Figure 3. A similar pattern of AMDP inhibition was observed with *Leptospira biflexa* K^+^-dependent H^+^-PPase (Artukka, E., Malinen, A.M., Baykov, A.A., unpublished).

The first molecule of each inhibitor is bound quite firmly to the free enzyme (*K*_i1_), and less strongly (but measurably) to the mono-substrate complex (*K*_i(s)_). Setting *A*_3_ to zero or setting *K*_i(s)_ to infinity (no mixed complex formed) made the fit worse for all inhibitors (Table S1). Assuming equal *K*_i1_, *K*_i2_, and *K*_i(s)_ values also resulted in an unsatisfactory fit (Table S1). Notably, the RMSD value did not exceed the mean error of rate measurements (6–10%) for the fits whose results are shown in Table 1, and the predicted rates showed no systematic deviations from the measured rates. 

### 2.2. Metal Cofactors Modulate Catalytic Asymmetry in mPPase

Earlier studies linked kinetic asymmetry to the alkali cation-binding site present in all Na^+^-transporting and K^+^-dependent H^+^-transporting mPPases [19]. Specifically, an Ala/Lys substitution introduced an alternative positive charge in this site and abolished kinetic cooperativity. Moreover, using a K^+^-deficient medium in the enzyme assay of different K^+^-dependent mPPases similarly abolished the kinetic cooperativity [19]. Here, we extend these findings by replacing K^+^ with Na^+^ in the cation-binding site of Dh-mPPase (Figure S2). This substitution did not change the substrate binding and inhibition patterns qualitatively (Figure S2), but augmented active site nonequivalence by increasing *K*_m2_ and decreasing *K*_m1_ and *K*_i1_ values (Table 1). These findings provide support for the role of the alkali cation-binding site in the catalytic asymmetry of mPPase. Furthermore, consistent with numerous earlier reports on various K^+^-dependent mPPases, Na^+^ was a less efficient activator in terms of *A*_1_ compared to K^+^ (Table 1).

**Figure 5:**
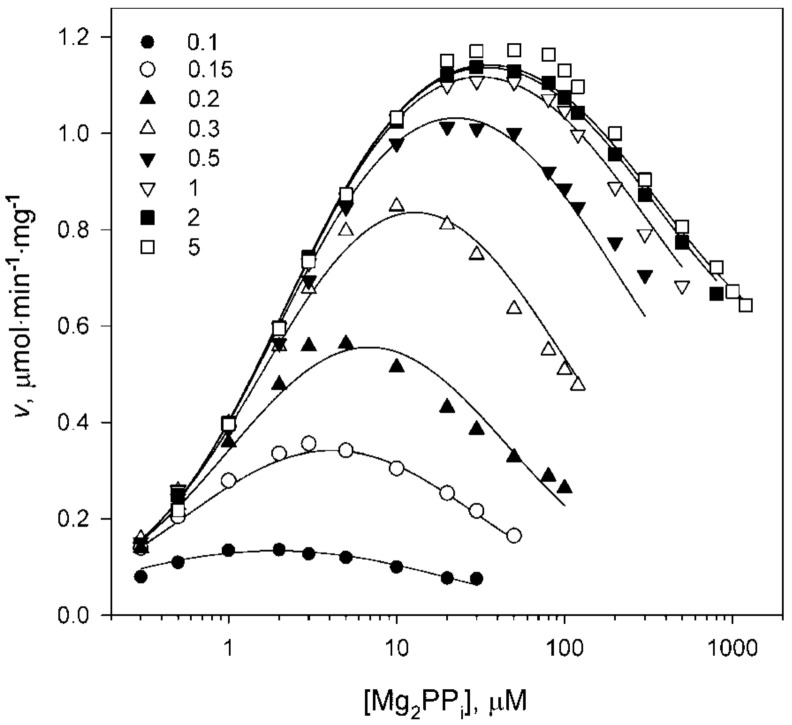
Kinetics of Dh-mPPase activation by Mg^2+^ in the presence of 50 mM K^+^. The abscissa shows the substrate concentration. Mg^2+^ concentrations (in mM) corresponding to different symbols are defined on the panel. The lines were calculated using Equation (3) with the parameter values found in Table 2.

**Figure 6:**
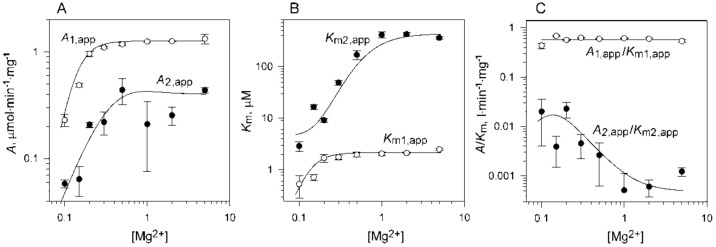
Secondary dependence of the kinetic parameters in Scheme 1 (**A**), maximal activities; (**B**), *K*_m_ values; (**C**), their ratios) on free Mg^2+^ concentration, as calculated from the data in Figure 5. All axes are scaled logarithmically. The lines were calculated using Equations (1) and (3a–d) with the parameter values found in Table 2.

The variation in free Mg^2+^ concentration in the assay medium dramatically affected the substrate concentration profile without changing its shape (Figure 5). The marked shift in the descending part of the curve to lower substrate concentrations indicated decreased *K*_m2_ value, whereas the decreased height indicated decreases in both *A*_1_ and *A*_2_. The apparent values of all parameters in Equation (1) were determined by fitting it to each dependence in Figure 5 measured at a fixed Mg^2+^ concentration. The best-fit values of *A*_1,app_, *A*_2,app_, *K*_m1,app_, and *K*_m2,app_ decreased with decreasing Mg^2+^ concentration in an unusually steep manner (Figure 6). Because of difficulties with saturating the second active site with the substrate, the errors in the parameters referring to the second active site were the highest for *A*_2,app_ and *K*_m2,app_. Notably, the ratio *K*_m2,app_/*K*_m1,app_ was relatively high at high Mg^2+^ concentrations, but approached 4—the value predicted for macroscopic constants of noninteracting sites [31]—at 0.1 mM Mg^2+^. 

The decline in *A*_1,app_ and *A*_2,app_ at low Mg^2+^ concentrations indicated Mg^2+^ release from the ES and SES complexes in Scheme 1. Relevant information on the substrate-free enzyme (E) was obtained from the analysis of *A*_1,app_/*K*_m1,app_ dependence (Figure 6C). The constancy of this composite parameter over the whole Mg^2+^ concentration range was a strong indication that the E species did not release or bind Mg^2+^ when its concentration was varied in the indicated ranges. 

**Scheme 3:**
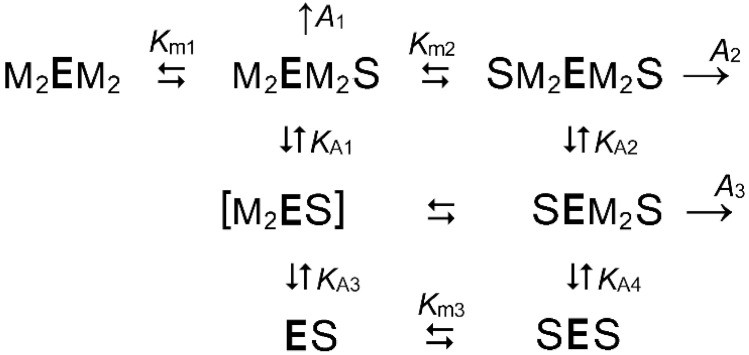
The kinetic model describing the activation of dimeric mPPase by Mg^2+^ in the presence of 50 mM K^+^. M is Mg^2+^, S is Mg_2_PP_i_, *K*_m1_–*K*_m3_ are macroscopic Michaelis constants, *K*_A1_–*K*_A4_ are macroscopic Mg^2+^-binding constants, *A*_1_–*A*_4_ are specific activities of the corresponding substrate-containing enzyme species. The bound S on the left and right sides of E refer to active sites in different subunits. Metal positioning in most species is arbitrary, as kinetic analysis reveals only the gross stoichiometry of the complexes. The species shown in brackets is stoichiometrically insignificant.

Various models, assuming release from one to four Mg^2+^ ions from the mono- and/or di-substrate complexes, were tried for the global fitting of the data in Figure 5. This analysis led to the model (Scheme 3) and the rate law (Equation (3)), which again are extended forms of Scheme 1 and Equation (1), with the apparent values of their parameters defined by Equation (3a–d). This model assumes that each of the ES and SES species of Scheme 1 releases four Mg^2+^ ions. Their release from (and binding to) the ES and SES species occurs in pairs, i.e., is highly cooperative within pairs, such that no intermediate species containing one to three Mg^2+^ ions are present in significant concentrations (all-or-none process). Furthermore, the binding of the two pairs of the metal to the ES demonstrated the high cooperativity between pairs, making the M_2_ES species also stoichiometrically insignificant, as described below. Numerical values of Scheme 3 parameters were determined by fitting Equation (3) to the rate data in Figure 5, using the values of *A*_1_, *A*_2_, *K*_m1_, and *K*_m2_ from Table 1 as initial estimates. The fitted values (Table 2) were not significantly different because the 5 mM Mg^2+^ concentration used in the inhibition studies (Figure 3) was nearly saturating. The values of *K*_A1_*K*_A3_ and *K*_A2_*K*_A4_ that characterize aggregated Mg^2+^-binding affinities of the mono- and di-substrate complexes differed 100-fold. If one keeps in mind that the magnesium complex of the free enzyme (M_2_EM_2_) does not dissociate, the difference between *K*_A1_*K*_A3_ and *K*_A2_*K*_A4_ means that stepwise substrate-binding progressively weakens Mg^2+^ binding to mPPase. Furthermore, the acquisition of the second substrate molecule makes Mg^2+^ release non-cooperative between pairs—values of *K*_A2_ and *K*_A4_ differ only 5-fold, not much different from the ratio of the macroscopic constant for binding to two noninteracting identical sites (see Section 4 Materials and Methods). Another important deduction is that the calculated value of *K*_m3_ that characterizes the binding of the second substrate molecule to SE is 100 times less than *K*_m2_, explaining the disappearance of the K-type cooperativity (*K*_m_ asymmetry) at low Mg^2+^ concentrations. Clearly, the magnesium cofactor induces this cooperativity via an increase in *K*_m2_, which was also evident from Figure 5. The omission of the M_2_ES species increased the root-mean-square deviation (RMSD) insignificantly (by 0.5%), and did not affect parameter values (Table 2), whereas the omission of an analogous SEM_2_S species increased RMSD by 80%, indicating the stoichiometric significance of the latter species. Scheme 3 without M_2_ES is thus the minimal scheme describing Dh-mPPase kinetics.

The model in Scheme 3, which is, of course, only an approximation of the complex equilibria in this system, is consistent with the available data on the Mg^2+^-binding stoichiometry. Each subunit of free enzyme in Scheme 3 contains two metal ions, based on the crystal structures of *Thermotoga maritima* mPPase in substrate-free and P_i_-bound forms [32,33]. Each bound substrate molecule adds two more Mg^2+^ ions, raising the number of bound Mg^2+^ ions to eight per dimer in the SM_2_EM_2_S complex. Indeed, the crystal structures of IDP-bound *V. radiata* and *T. maritima* mPPases contain ten Mg^2+^ ions per dimer [17,25,33], but two of them appear to be an artifact of the crystallization procedure [33].

### 2.3. Membrane Role in Catalytic Asymmetry

Our data reported here and previously [19,21] indicate negative kinetic cooperativity in interaction with the substrate—*K*_m2_ greatly exceeds *K*_m1_. However, two other groups recently reported positive cooperativity for mPPases assayed in a solubilized form [25] or in yeast vesicles [11]. All groups agree, however, that *A*_1_ exceeds *A*_2_. As we are confident of our activity assay, we sought out a less trivial explanation of this discrepancy. In view of the growing body of evidence for the importance of lipids in the mechanisms of membrane proteins, including allostery [34], we measured the substrate saturation profile for DDM-solubilized Dh-mPPase and compared it with the profile for the IMV-bound enzyme (the uppermost curves in Figure 3 and Figure 5). This comparison demonstrated the close similarity of the two profiles (Figure S3) and the parameters derived therefrom (Table S2). Only a twofold decrease in the *K*_m2_/*K*_m1_ ratio was observed in the DDM-solubilized mPPase. This result indicated that the catalytic asymmetry of mPPase is an inherent property of this enzyme rather than the effect of the membrane, which is only modulatory.

## 3. Discussion

### 3.1. Catalytic Asymmetry in mPPase

Earlier studies demonstrated that substrate binding to one active site of homodimeric mPPase increased the Michaelis constant (K-type cooperativity) and decreased the catalytic constant (V-type cooperativity) for the other active site [19,20]. Here, we have determined the binding scheme for three non-hydrolyzable PP_i_ analogs, which acted as competitive inhibitors in PP_i_ hydrolysis, and the effects of their binding on catalysis in the other active site. This analysis was conducted in a wide range of substrate (Mg_2_PP_i_) and inhibitor concentrations to allow the formation of the mixed complex (SEI in Scheme 2) in addition to the enzyme complexes with only a substrate or an inhibitor. Based on the parameter values of Scheme 2 (Table 1), the maximal fraction of the IES species varied from 26% with IDP to 59% with AMDP at the highest inhibitor concentrations used (Figure S4), making this species stoichiometrically significant in our kinetic analyses. 

Our kinetic data provide two lines of evidence in support of the functional asymmetry in mPPase. First, the inhibition constants for inhibitor binding to two active sites in dimeric mPPase, *K*_i1_ and *K*_i2_, differed significantly, for IDP and HEDP in particular (Table 1), indicating negative binding cooperativity. This difference was of the same magnitude as that between *K*_m1_ and *K*_m2_ values. In other words, the inhibitor or substrate binding to one active site impairs their interaction with the second site. Interestingly, the heterotrophic effects are an order of magnitude smaller—inhibitor binding to one site increases *K*_m_ for the second site by a factor of 10–20 (compare *K*_m1_ and *K*_m(i)_ in Table 1). As a result, *K*_m(i)_ is intermediate between *K*_m1_ and *K*_m2_, indicating that substrate and inhibitor exert different changes in the structure of the neighboring subunit. The substrate equally affected inhibitor binding (compare *K*_i1_ and *K*_i(s)_ in Table 1) because the four constants are interrelated: *K*_m1_*K*_i(s)_ = *K*_i1_*K*_m(i)_. The negative binding cooperativity was the smallest with AMDP (*K*_i2_/*K*_i1_ = 31). 

A comparison between the activity values *A* in Table 1 shows that the occupancy of the second site, whether by substrate or inhibitor, caused inhibition, providing additional support for the active site interdependence. The values of *A*_1_ and *A*_2_ differ approximately threefold, but, keeping in mind that *A*_1_ refers to one and *A*_2_ refers to two active sites, the actual substrate inhibition was two times greater, i.e., sixfold. In contrast, the values of *A*_1_ and *A*_3_ are directly comparable because both parameters refer to the enzyme with only one active site working. They differ less with IDP and HEDP—only 2.5-fold—again indicating that substrate and inhibitor change the structure of the neighboring subunit differently. With AMDP, *A*_1_ and *A*_3_ differed sixfold, similar to the *A*_1_ and *A*_2_ values. 

A note on the activity assay is appropriate because its accuracy/precision was essential for deriving reliable reaction schemes containing multiple enzyme species. Because the assay was continuous and sensitive, it allowed a reliable estimation of initial velocity at a submicromolar Mg_2_PP_i_ concentration [28], as strictly required by the low *K*_m1_ value. The lowest total PP_i_ concentration (corresponding to 0.5 µM Mg_2_PP_i_ at 5 mM Mg^2+^) was 0.79 µM, which yielded 1.58 µM P_i_ and produced a 7 cm signal on recorder paper upon complete hydrolysis. Several precautions were taken to ensure the validity of the measured initial velocity rates at high substrate concentrations. Specifically, we considered the decreased sensitivity of the P_i_ assay in the presence of PP_i_ and HEDP and limited the substrate concentration to its solubility range. 

In addition, our analyses posited Mg_2_PP_i_ as the actual substrate, but one should understand that this is an arbitrary choice. It is not generally appreciated that steady-state kinetic studies can define only the stoichiometry of the statistically significant enzyme species, and not the ways of their formation. For instance, the six Mg^2+^ ions per dimer of the species SEM_2_S in Scheme 3 (S = Mg_2_PP_i_) can be distributed differently between two active sites, but this will not change the rate equation. Furthermore, because the ratio of Mg_2_PP_i_ and MgPP_i_ concentrations at a fixed Mg^2+^ concentration equals [Mg^2+^]/*K*_M2L_ and is therefore constant, assuming MgPP_i_ to be the actual substrate for Schemes 1 and 2 would only change the values of *K*_m1_, *K*_m2_, and *K*_m(i)_, without affecting their ratios. In reality, all PP_i_ species, including free PP_i_, may be substrates, i.e., may bind to the enzyme–magnesium complexes of different stoichiometries. The complexity of the PP_i_–Mg^2+^–K^+^/Na^+^ equilibria in the mPPase assay mixture thus creates a certain ambiguity in data interpretation. Nevertheless, using the species present in the assay mixture (Mg^2+^ and Mg_2_PP_i_ or MgPP_i_) as independent variables greatly simplifies the kinetic analysis by comparison with the approach relying on total concentrations of PP_i_ and magnesium. 

### 3.2. The Roles of the Metal Cofactors in the Catalytic Asymmetry

Our new data indicate that two metal cofactors, Mg^2+^ and K^+^, control the active site asymmetry in the interaction with substrate and its analogs. Interestingly, Mg^2+^ affected only the K-type cooperativity, which decreased with decreasing Mg^2+^ concentration and vanished at 0.1 mM Mg^2+^. This effect resulted solely from a change in *K*_m2_ (Figure 6B). In contrast, the V-type cooperativity was Mg^2+^-independent—although both *A*_1_ and *A*_2_ decreased at low [Mg^2+^] (Figure 6A), their ratio did not change significantly. The effect of K^+^ replacement by Na^+^ on the *K*_m2_/*K*_m1_ ratio (Table 1) suggests a role for the alkali–cation binding center in kinetic cooperativity, consistent with the non-cooperative behavior of several mPPases in the absence of an alkali metal cofactor [19]. 

Scheme 3, derived from the kinetic data, indicates that catalysis requires four Mg^2+^ ions per active site. Two are parts of the actual substrate, Mg_2_PP_i_, and two are prebound by the enzyme, consistent with the X-ray crystallographic data [17,25,32,33]. The two Mg^2+^ ions that control the K-type cooperativity in mPPase are bound with the lowest affinity in the enzyme–substrate complex. Based on the crystal structure of the *V. radiata* mPPase complexed with IDP [17], they likely are substrate metals, which have fewer stabilizing contacts with the enzyme. 

Most previous kinetic studies [35,36,37] addressed the role of Mg^2+^ ions in mPPase by considering only the rising part of the substrate concentration dependence of activity, controlled by *K*_m1_. In one of them, highly cooperative Mg^2+^ binding to the substrate complex of Na^+^-transporting mPPase of *Methanosarcina mazei* was observed [37], similar to that seen in the current study. There was only one publication in which the effects of Mg^2+^ on the hydrolysis kinetics of a different H^+^-transporting mPPase (from *Chlorobium limicola*) were analyzed in a wide range of substrate concentrations, allowing substrate binding to both active sites [21]. This mPPase belongs to a highly diverged subfamily of mPPases that are regulated by Na^+^ and K^+^ ions. The *C. limicola* enzyme exhibited a similar active site nonequivalence, but a smaller Mg^2+^-binding affinity in the substrate-free state, and much higher Mg^2+^-binding affinity in the substrate-bound state in the absence of Na^+^. Accordingly, the observed effects on the activity in the same Mg^2+^ concentration range were relatively small. In the presence of 100 mM Na^+^, the effects were much more prominent because Na^+^ competed with Mg^2+^ for binding to ES. In these conditions, the effects of Mg^2+^ on kinetic cooperativity differed from those in Dh-mPPase—K-type cooperativity increased with decreasing Mg^2+^ concentration, whereas V-type cooperativity changed from negative to positive. Altogether, the available data suggest that Mg^2+^ binding universally controls kinetic cooperativity in mPPases, but in different ways, depending on the relative Mg^2+^-binding affinities of different enzyme forms.

## 4. Materials and Methods

### 4.1. Materials

Inverted membrane vesicles (IMV) containing Dh-mPPase were prepared from *Escherichia coli* C41(DE3)ril cells expressing the Dh-mPPase gene as described previously [19]. IMV were suspended in storage buffer (10 mM MOPS-KOH (pH 7.2), 900 mM sucrose, 5 mM DTT, 1 mM MgCl_2_, and 50 µM EGTA), and aliquots were frozen in liquid nitrogen and stored at −80 °C. Vesicles were quantified by determining their protein content using the Bradford assay [38]. 

To extract Dh-mPPase from IMV, 30 µL of IMV suspension (0.9 mg protein) were diluted with 350 µL of cold solubilization buffer (50 mM MOPS-KOH, 200 mM KCl, 0.5 mM MgCl_2_, 1 mM dithiothreitol, 0.5 mM tetrasodium IDP, 20% glycerol, and 150 mM sucrose, pH 7.2). *n*-Dodecyl-β-d-maltoside (3 mg dissolved in 20 µL of the solubilization buffer) was slowly added in 2-µL aliquots with gentle mixing after each addition. The mixture was incubated on ice for 1 h with occasional mixing and centrifuged at 17,000× *g* for 1 h. The clear supernatant containing solubilized Dh-mPPase was used for activity measurements.

As K^+^ and Na^+^ are competing cofactors of Dh-mPPase, precautions were taken to exclude Na^+^ from K^+^-containing assay mixtures. To this end, tetrasodium PP_i_ (Sigma-Aldrich, Saint Louis, MO, USA) was converted into its tetrapotassium form by passing through a column with Dowex 50W X8 charged with K^+^. Potassium salts of HEDP (etidronic acid) and AMDP were prepared by neutralizing their free acids (gifts of N. M. Dyatlova and S. V. Komissarenko) with KOH. IDP was synthesized as described [39] and used as authentic tetrasodium salt in all cases because 0.2 mM Na^+^, maximally added with IDP, had a negligible effect on the enzyme activity. 

### 4.2. PP_i_ Hydrolysis Assay

Because *E. coli* contains only soluble pyrophosphatase, which is washed away during IMV isolation (<1% remaining background activity, as determined with *E. coli* transformed with empty plasmid), the IMV containing Dh-mPPase could be used directly for activity measurements. The activity assay medium (typically 25 mL in volume) contained 0.1 M MOPS-KOH buffer, pH 7.2, and varied concentrations of free Mg^2+^ ion (added as MgCl_2_), Mg_2_PP_i_ complex, and other indicated additions. Reactions were initiated by adding IMV suspension (0.01–0.24 mg protein), and P_i_ accumulation due to PP_i_ hydrolysis was continuously recorded for 2–3 min at 25 °C using an automated P_i_ analyzer [28] at a sensitivity of 4–20 µM P_i_ per recorder scale. The IMV amount was varied to achieve similar measured rates at different substrate, inhibitor, or Mg^2+^ concentrations and, hence, the equal relative error of measurement for each data point [28]. The measured rate values were then normalized to the same IMV amount by calculating specific activities. Appropriate corrections were made for a linear decrease in the sensitivity of the P_i_ assay with increasing PP_i_ and HEDP concentrations (Figure S1). The effect of PP_i_ is explained by the competitive formation of 12-molibdopyrophosphoric acid, demonstrated directly by Raman spectroscopy [40] and indirectly by the formation of blue species in the colorimetric assay for PP_i_ [41]. The rate values obtained in replicate measurements agreed within 5–10%, and were proportional to the enzyme concentration. 

### 4.3. Calculations and Data Treatment

The total concentrations of MgCl_2_ and PP_i_ required to maintain the desired concentrations of Mg_2_PP_i_ complex (presumed actual substrate [20,35,42]) and free Mg^2+^ ion in the activity assay mixture at pH 7.2 were calculated using the apparent dissociation constants for the MgPP_i_ and Mg_2_PP_i_ complexes listed in Table 3. These apparent constants were calculated from the stabilities of individual complexes formed between PP_i_, H^+^, Mg^2+^, K^+^, and Na^+^, as described before [28]. The same algorithm was used to calculate the apparent dissociation constants for MgHEDP and Mg_2_HEDP complexes at pH 7.2 in the presence of 50 mM K^+^ from comparable data on the individual complexes formed by this PP_i_ analog [43]. These apparent constants (Table 3) were used to calculate the composition of the assay medium in the experiments measuring HEDP effects. Mg^2+^ sequestration by IDP and AMDP was ignored because of the low concentrations used (50 and 20 µM at maximum) compared to the Mg^2+^ concentration (5 mM). Our analysis of rate dependence used Mg_2_PP_i_ (substrate) and free Mg^2+^ (activator) concentrations as the independent variables.

Non-linear least-squares fittings were performed using the program Scientist (MicroMath). Rate (*v*) values were weighed according to 1/*v*^2^ because the error in *v* is proportional to the *v* value (equal relative errors) for our assay setup, which included the variation in the enzyme amount to ensure similar measured rates for all data points.

The substrate dependence of enzyme activity was analyzed in terms of Scheme 1, which assumes that the substrate binding to one active site affects catalysis in the other active site in the dimeric Dh-mPPase.

Equation (1) gives the rate equation for Scheme 1, where [S] is substrate concentration. When fitting Equation (1) to the bell-shaped dependencies of *v* on [S], the maximum rate, the rate at maximal [S], and [S] values corresponding to half-effects on the ascending and descending parts were used as initial estimates of the parameters *A*_1_, *A*_2_, *K*_m1_, and *K*_m2_, respectively.

*v* = (*A*_1_ + *A*_2_[S]/*K*_m2_)/(1 + *K*_m1_/[S] + [S]/*K*_m2_) (1)

Scheme 1 expands to Scheme 2 for the reaction occurring in the presence of non-hydrolyzable substrate analogs, which compete with the substrate for the active sites. Scheme 2 includes an additional catalytically competent mixed complex (IES) containing substrate and inhibitor in different subunits of the enzyme molecule. 

The rate equation for Scheme 2 is given by Equation (2),

*v* = (*A*_1_ + *A*_2_[S]/*K*_m2_ + *A*_3_[I]/*K*_i(s)_)/{1 + *K*_m1_(1 + [I]/*K*_i1_ + [I]^2^/*K*_i1_*K*_i2_)/[S] + [S]/*K*_m2_) + [I]/*K*_i(s)_}, (2)

which was derived assuming steady-state kinetics for the reaction with the substrate and equilibrium kinetics for inhibitor binding, a common practice in inhibition studies [31] (pp. 98–101). Equation (2) can be rearranged into Equation (1) with the apparent parameters defined by Equation (2a–d):

*A*_1,app_ = (*A*_1_ + *A*_3_[I]/*K*_i(s)_)/(1 + [I]/*K*_i(s)_) (2a)

*K*_m1,app_ = *K*_m1_{1 + [I]/*K*_i1_ + [I]^2^/(*K*_i1_*K*_i2_)}/(1 + [I]/*K*_i(s)_) (2b)

*A*_2,app_ = *A*_2_/(1 + [I]/*K*_i(s)_) (2c)

*K*_m2,app_ = *K*_m2_(1 + [I]/*K*_i(s)_) (2d)

For simplicity, Scheme 1 and Scheme 2 are written in terms of macroscopic Michaelis and inhibitor-binding constants, which do not differentiate between identical sites in oligomeric proteins, in contrast to microscopic constants, which refer to the particular sites. For a homodimeric protein, the microscopic and macroscopic constants are linked by the following relationships: *K*_x1(micro)_ = 2*K*_x1(macro)_, *K*_x2(micro)_ = 0.5*K*_x2(macro)_, where x = m or i [31] (p. 17). Accordingly, *K*_x2(micro)_ = *K*_x1(micro)_ and *K*_x2(macro)_ = 4*K*_x1(macro)_ for non-interacting sites. These relationships are not satisfied for Dh-mPPase (*K*_m2(macro)_ >> 4*K*_m1(macro)_), demonstrating strong negative kinetic cooperativity [19].

Scheme 3 is a further extension of Scheme 1 for the enzymatic reaction occurring at varied substrate and Mg^2+^ concentrations. Scheme 3 assumes that catalysis requires binding of two Mg^2+^ ions per subunit in addition to the two Mg^2+^ ions bound to PP_i_ to form the true substrate. 

The rate equation for Scheme 3 is given by Equation (3), which reduces to Equation (1) with the parameter values defined by Equation (3a–d):

*v* = {*A*_1_ + *A*_2_[S]/*K*_m2_ + *A*_3_*K*_A2_[S]/(*K*_m2_[M]^2^)}/{1 + *K*_m1_/[S] + *K*_A1_*K*_A3_/[M]^4^ + [S](1 + *K*_A2_/[M]^2^ + *K*_A2_*K*_A4_/[M]^4^)/*K*_m2_} (3)

*A*_1,app_ = *A*_1_/(1 + *K*_A1_/[M]^2^ + *K*_A1_*K*_A3_/[M]^4^) (3a)

*K*_m1,app_ = *K*_m1_/(1 + *K*_A1_/[M]^2^ + *K*_A1_*K*_A3_/[M]^4^) (3b)

*A*_2,app_ = (*A*_2_ + *A*_3_*K*_A2_/[M]^2^)/(1 + *K*_A2_/[M]^2^ + *K*_A2_*K*_A4_/[M]^4^) (3c)

*K*_m2,app_ = *K*_m2_(1 + *K*_A1_/[M]^2^ + *K*_A1_*K*_A3_/[M]^4^)/(1 + *K*_A2_/[M]^2^ + *K*_A2_*K*_A4_/[M]^4^) (3d)

## 5. Conclusions and Perspectives

**Figure 7:**
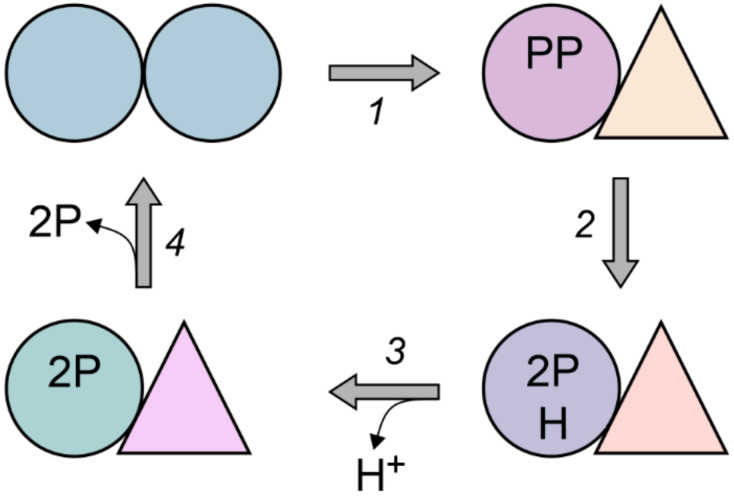
A hypothetical model of the conformationally coupled PP_i_ hydrolysis and H^+^ transport in dimeric mPPase. PP is pyrophosphate, P is phosphate, and H is proton. Subunits are shown as different geometrical shapes and colors, which characterize their structure and energy state.

To sum up, the new data support and further develop the notion that the two active sites cooperate in mPPase catalysis. The results of our investigation show that substrate or substrate analog binding to subunit A distorts the structure of the subunit B active site, impeding its substrate binding and conversion ability. We speculate that the binding energy is thus stored as a conformational strain. We hypothesize that the stored energy is back-used to remove products from subunit A and return its active site into the resting state. According to Shah et al. [44], substrate-binding affects active site closure via the loop connecting α-helices 5 and 6 in mPPase. After substrate hydrolysis and ion transport, the active site should be unclosed for the next catalytic cycle. The complete sequence of events in H^+^ transport could be as follows: (a) substrate binds to active site A, resulting in a conformational change (site closure) that is propagated to active site B in the other subunit; (b) the substrate is attacked by the activated water molecule located at the entrance to the ion-conducting channel. This reaction yields two phosphate molecules that remain bound in the active site and a proton, which creates high local acidity; (c) the proton moves via a water wire to the other side of the membrane down the locally favorable proton potential gradient; (d) the loop lid opens because its weaker interactions with the two phosphates cannot offset the conformational strain, and the phosphates leave the active site. This sequence of events is shown schematically in Figure 7, where different shapes correspond to different subunit structures and energy states. The scheme assumes that the binding energy is accumulated in the right subunit in step 1 and released in step 4. Alternatively, the stored energy may drive H^+^ transport in the left subunit (step 3). 

The enzyme dimer thus performs asymmetrically—one subunit carries out PP_i_ hydrolysis and ion transport while the other acts as a transient energy store. These roles of the two subunits can interchange in the next catalytic cycle. The fact that Mg^2+^ ions increase the asymmetry is explained by their essential role in substrate binding—their interactions with the enzyme, including the loop lid, are mostly through Mg^2+^ ions [17,33]. Accordingly, the lower the number of Mg^2+^ ions that are bound, the weaker the conformational strain will be upon substrate binding. Similar arguments can explain the effects of substrate binding on the Mg^2+^-binding cooperativity and binding affinity. Our model of mPPase catalysis differs in three major points from that proposed by Vidilaseris et al. [25]. First, the two models assume different roles for the conformational energy evolved in the PP_i_ binding step and the chemical energy evolved in the PP_i_ hydrolysis step. Vidilaseris et al. assumed that these energies are used, respectively, for proton transport and product release, because they believed that transport precedes hydrolysis. Based on the reinterpretation [18] of the electrometric data reported by Goldman’s group [33,44], our model presumes that the transport event follows PP_i_ hydrolysis and accordingly ascribes interchanged roles for the conformational and chemical energy. Second, our model does not require PP_i_ binding to both subunits because such binding decreases the catalytic efficiency of mPPase, and can hardly be significant at the PP_i_ concentrations expected in the cell. Third, Vidilaseris et al. assumed that the conformational energy is immediately used in the same subunit rather than stored in the other subunit. These differences mainly arise from different coupling principles on which the two models rely. Unlike the model of Vidilaseris et al. [25], our model assumes “direct” coupling, the principle widely used by transporting oxidoreductases, wherein the transported charge (proton or electron) is the one generated in the coupled chemical reaction [45]. 

The proposed “oscillatory” mechanism of mPPase, the “rotary” mechanism of F-ATPase, and the ones of other ion-transporting ATPases resemble each other in using the conformational energy to couple ion transport with polyphosphate hydrolysis. mPPase appears to be an evolutionary progenitor of these transporters, which retained the part of the mPPase mechanism related to conformational energy. However, the higher energy cost of ATP compared with PP_i_ necessitates more elaborate machinery with separate hydrolytic and ion-transporting parts, and, consequently, a more complex subunit structure. Besides pointing to their mechanistic similarity with the other transporters, our findings have raised several structure-related questions to answer in future studies. How does substrate binding in one subunit interfere with its binding to the other subunit? How is this mechanism governed by the metal ions of the other subunit? What is the structure of the dimer having substrate in only one subunit? What structure elements are involved in subunit communication? These questions are addressed in the structural study currently underway.

## Figures and Tables

**Table 1:** Kinetic parameters for Dh-mPPase inhibition by substrate analogs according to Scheme 2. Free Mg^2+^ concentration was maintained at 5 mM, and substrate (Mg_2_PP_i_) concentration was varied in the range of 0.5–1100 µM. The alkali metal cofactor was 50 mM K^+^, except for the column marked “IDP (Na),” for which it was 50 mM Na^+^. The *K*_i_ values are shown in terms of total concentrations of the substrate analogs.

Parameter	Value			
	IDP	HEDP	AMDP	IDP (Na)
*A*_1_ (µmol·min^−1^·mg^−1^)	1.32 ± 0.01	1.31 ± 0.02	1.34 ± 0.02	0.27 ± 0.01
*A*_2_ (µmol·min^−1^·mg^−1^)	0.40 ± 0.04	0.37 ± 0.04	0.49 ± 0.02	<0.20
*A*_3_ (µmol·min^−1^·mg^−1^)	0.53 ± 0.05	0.51 ± 0.02	0.23 ± 0.03	0.07 ± 0.04
*K*_i1_ (µM)	4.2 ± 0.2	27 ± 1	0.40 ± 0.02	2.2 ± 0.2
*K*_i2_ (µM)	>500	>10,000	12.5 ± 2.5	>500
*K*_i(s)_ (µM)	90 ± 10	470 ± 30	4.4 ± 0.4	110 ± 30
*K*_m1_ (µM)	2.5 ± 0.1	2.3 ± 0.1	2.6 ± 0.1	1.4 ± 0.1
*K*_m2_ (µM)	460 ± 60	490 ± 50	280 ± 30	>1000
*K*_m(i)_ * (µM)	53 ± 6	40 ± 3	27 ± 3	70 ± 20
RMSD (%)	3.46	3.62	3.39	7.08

* Dependent parameter calculated as *K*_m1_*K*_i(s)_/*K*_i1_.

**Table 2:** Kinetic parameters for Dh-mPPase activation by Mg^2+^ as calculated from the data in Figure 5 according to Scheme 3 and its shorter variant.

Parameter	Value	
	Scheme 3	Scheme 3 without M_2_ES
*A*_1_ (µmol·min^−1^·mg^−1^)	1.27 ± 0.02	**1.27 ± 0.02**
*A*_2_ (µmol·min^−1^·mg^−1^)	0.40 ± 0.06	**0.40 ± 0.05**
*A*_3_ (µmol·min^−1^·mg^−1^)	0.60 ± 0.05	**0.62 ± 0.05**
*K*_A1_ (µM^2^)	0.004 ± 0.002	**-**
*K*_A2_ (µM^2^)	0.47 ± 0.09	**0.53 ± 0.09**
*K*_A3_ (µM^2^)	0.17 ± 0.12	**-**
*K*_A4_ (µM^2^)	0.10 ± 0.01	**0.10 ± 0.01**
*K*_A1_*K*_A3_ (µM^4^)	0.00057 ± 0.00002	**0.00060 ± 0.00002**
*K*_A2_*K*_A4_ (µM^4^)	0.05 ± 0.01	**0.05 ± 0.01**
*K*_m1_ (µM)	2.2 ± 0.1	**2.17 ± 0.05**
*K*_m2_ (µM)	420 ± 70	**430 ± 80**
*K*_m3_ * (µM)		**5 ± 1**
RMSD (%)	3.80	**3.82**

* Dependent parameter calculated as *K*_m2_*K*_A1_*K*_A3_/(*K*_A2_*K*_A4_).

**Table 3:** The apparent dissociation constants for PP_i_ and HEDP complexes with Mg^2+^ at pH 7.2, 25 °C, as calculated from the literature’s data [28,43]. *K*_ML_ and *K*_M2L_ refer to mono-magnesium and dimagnesium complexes of PP_i_ or HEDP, respectively.

Ligand	Alkali Metal Ion (50 mM)	Dissociation Constant, mM	
		*K*_ML_	*K*_M2L_
PP_i_	K^+^	0.112	2.84
PP_i_	Na^+^	0.122	2.84
HEDP	K^+^	0.200	1.79

## Data Availability

Not applicable.

## Supplementary Materials

The following are available online at www.mdpi.com/article/10.3390/ijms22189820/s1, Figure S1: Pyrophosphate decreases the sensitivity of the phosphate assay, Figure S2: Kinetics of Dh-mPPase inhibition by IDP in the presence of 50 mM Na^+^ as an alkali metal cofactor, Figure S3: Substrate saturation profiles for Dh-mPPase in the IMV-bound form (open circles) and in the DDM-solubilized form (closed circles), Figure S4: The distribution of different enzyme species in Scheme 2 as a function of substrate concentration at the maximal PP_i_ analog concentrations used (50 µM IDP, 1000 µM HEDP, or 20 µM AMDP), Table S1: Comparison of reduced versions of Scheme 2 in terms of fit goodness, as characterized by the RMSD value, Table S2: Parameters values for Scheme 1, derived from the profiles shown in Figure S3.
