# Supplementary material for: Catalytic Asymmetry in Homodimeric H+-Pumping Membrane Pyrophosphatase Demonstrated by Non-Hydrolyzable Pyrophosphate Analogs
Source: Int J Mol Sci. 2021 Sep 10;22(18):9820. doi: 10.3390/ijms22189820 (PMC8469034; doi:10.3390/ijms22189820)
Supplement: Supplementary file 1 [file ijms-22-09820-s001.zip › ijms-1349216-supplementary.pdf]

Supplementary Material for:

"Catalytic asymmetry in homodimeric H^+-pumping membrane pyrophosphatase demonstrated by non-hydrolyzable pyrophosphate analogs"

Viktor A. Anashkin, Anssi M. Malinen, Alexander V. Bogachev, and Alexander A. Baykov

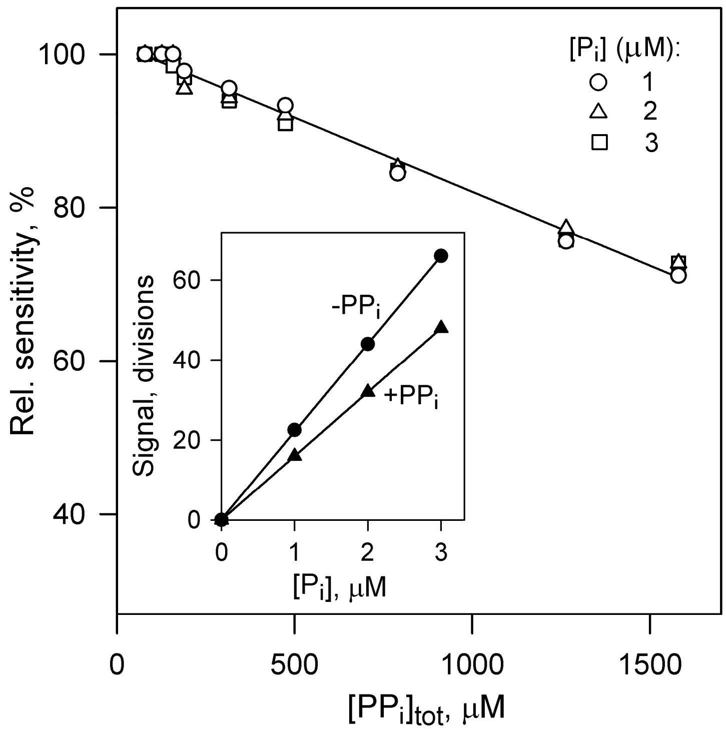

Figure S1. Pyrophosphate decreases the sensitivity of the phosphate assay. The dependence of the relative size of the signal produced by 1, 2, or 3 μM phosphate on total PP_i concentration is shown. All signals were corrected for the background values obtained without phosphate. Full-scale recorder signal (100 divisions) corresponded to an A_{660} change of 0.05 in the measuring cuvette of the phosphate analyzer. The *inset* shows linear phosphate calibration curves obtained in the absence or presence of 1.6 mM total pyrophosphate. Samples additionally contained 5 mM $MgCl_2$ (*-PP_i* curve) or 7.6 mM $MgCl_2$ (*+PP_i* curve) and 0.1 M MOPS/KOH buffer, pH 7.2. $MgCl_2$ (up to 10 mM) did not affect the P_i assay sensitivity. HEDP effect on P_i assay was similar but 1.8 times less (1 mM HEDP decreased the signal by 10 % independently of the substrate concentration).

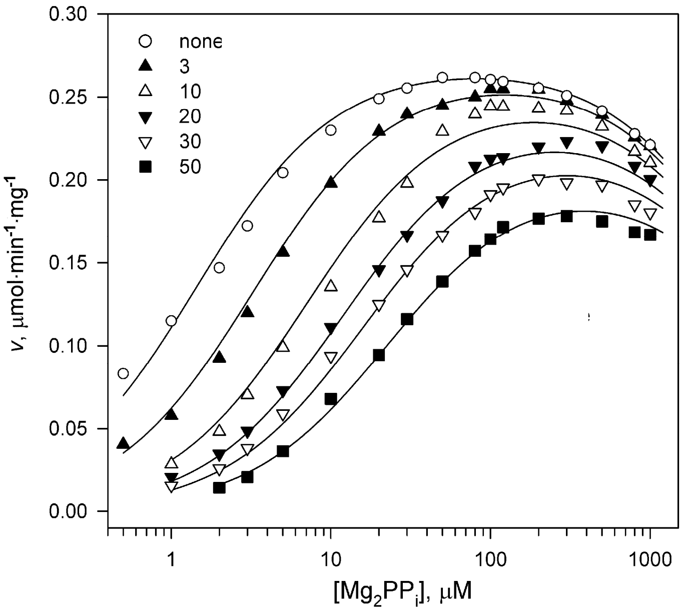

Figure S2. Kinetics of Dh-mPPase inhibition by IDP in the presence of 50 mM Na^+ as an alkali metal cofactor. The ordinate shows substrate (Mg_2PP_i) concentration and is scaled logarithmically. IDP concentrations (in µM) corresponding to different symbols are defined on the panel.

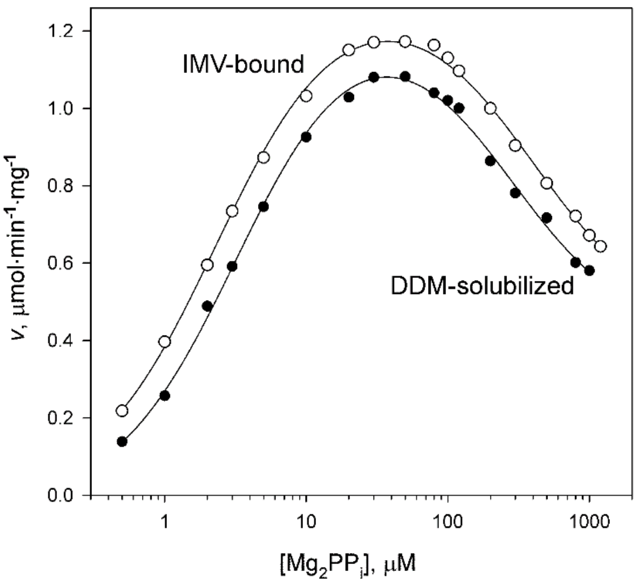

Figure S3. Substrate saturation profiles for Dh-mPPase in the IMV-bound form (open circles) and in the DDM-solubilized form (closed circles). Free Mg^{2+} concentration was 5 mM. The data for the IMV-bound enzyme were the same as in Fig. 2 (in the absence of the inhibitors) and Fig. 4 (at 5 mM Mg^{2+}). The lines were obtained with Eq 1 using the best-fit parameter values found in Table S2.

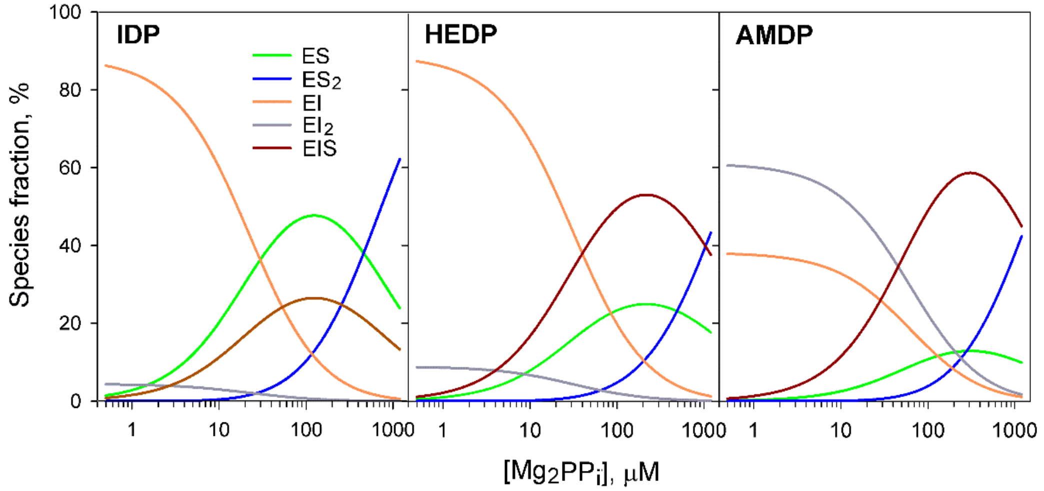

Figure S4. The distribution of different enzyme species in Scheme 2 as a function of substrate concentration at the maximal PP_i analog concentrations used (50 μM IDP, 1000 μM HEDP, or 20 μM AMDP). The distribution was calculated using the parameter values found in Table 1.

Table S1. Comparison of reduced versions of Scheme 2 in terms of fit goodness, as characterized by the RMSD value

Assumption	Omitted species	RMSD (%)		
		IDP	EHDP	AMDP
None		3.46	3.63	3.39
$K_{i2} = \infty$	IEI	3.46	3.62	3.94*
$K_{i2} = \infty$, $A_3 = 0$	IEI	3.88	7.50	4.03
$K_{i(s)} = \infty$	IES	5.99	9.76	11.0
$K_{i1} = K_{i2} = K_{i(s)}$		5.79	8.19	8.6

* The fitting procedure generated A_3 of 0.03 ± 0.01 μmol·min^{-1}·mg^{-1} for this sub-model.

Table S2. Parameters values for Scheme 1, derived from the profiles shown in Fig. S3

Parameter	Value	
	IMV-bound enzyme	DDM-solubilized enzyme
A_1 (μmol·min^{-1}·mg^{-1})	1.34 ± 0.02	1.32 ± 0.03
A_2 (μmol·min^{-1}·mg^{-1})	0.44 ± 0.03	0.43 ± 0.04
K_{m1} (μM)	2.4 ± 0.1	3.3 ± 0.2
K_{m2} (μM)	380 ± 40	270 ± 50
